# Supplementary material for: High-density, highly sensitive sensor array of spiky carbon nanospheres for strain field mapping
Source: Nat Commun. 2024 May 4;15:3752. doi: 10.1038/s41467-024-47283-8 (PMC11069524; doi:10.1038/s41467-024-47283-8)
Supplement: Supplementary file 1 — Supplementary information [file 41467_2024_47283_MOESM1_ESM.pdf]

Supplementary information

High-density, highly sensitive strain array of spiky carbon nanospheres for strain field mapping

Shuxing Mei[1]†, Haokun Yi[1]†, Jun Zhao[1], Yanting Xu[1], Lan Shi[1], Yajie Qin[2], Yizhou Jiang[2], Jiajie Guo[3], Zhuo Li[1*] and Limin Wu [1*]

[1]Department of Materials Science and State Key Laboratory of Molecular Engineering of Polymers, Fudan University, 220 Handan Rd., Shanghai 200433, China.

[2]Micro-Nano System Center, School of Information Science and Technology, Fudan University, 220 Handan Rd., Shanghai 200433, China.

[3]State Key Laboratory of Intelligent Manufacturing Equipment and Technology, School of Mechanical Science and Engineering, Huazhong University of Science and Technology, Wuhan, Hubei, China

† These authors contributed equally: Shuxing Mei, Haokun Yi.

*Corresponding authors: lmw@fudan.edu.cn (L. W.); zhuo_li @ fudan.edu.cn (Z. L.).

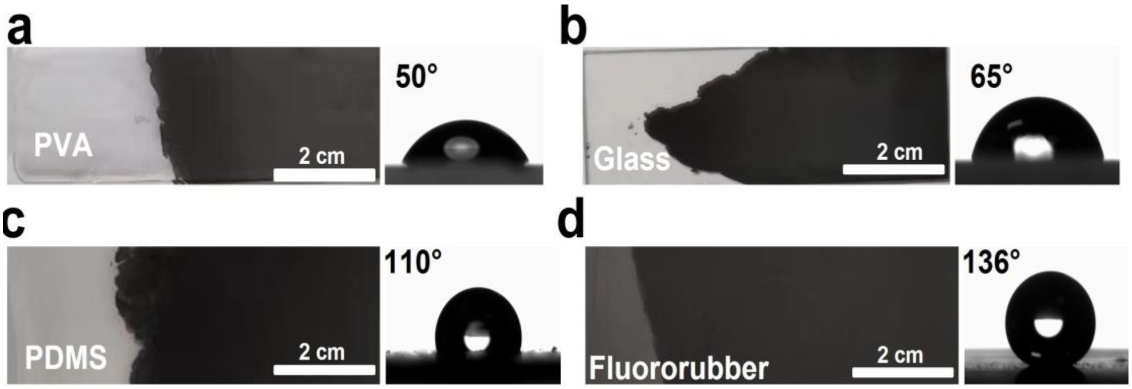

Supplementary Figure 1. SCN films transferred from the water surface onto different substrates and the water contact angle of different substrates. (a) PVA (polyvinyl alcohol); (b) glass; (c) PDMS (polydimethylsiloxane); (d) Fluororubber.

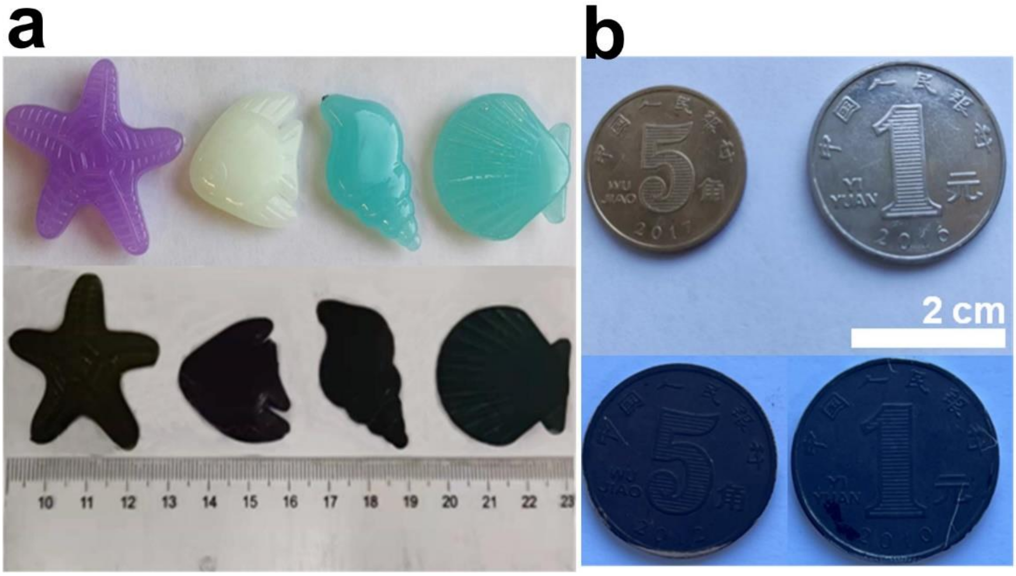

Supplementary Figure 2. Conformal coverage of the SCN films on complex surfaces. (a) Children's toys with complex surface topology. (b) 5-cent and 1-yuan coins.

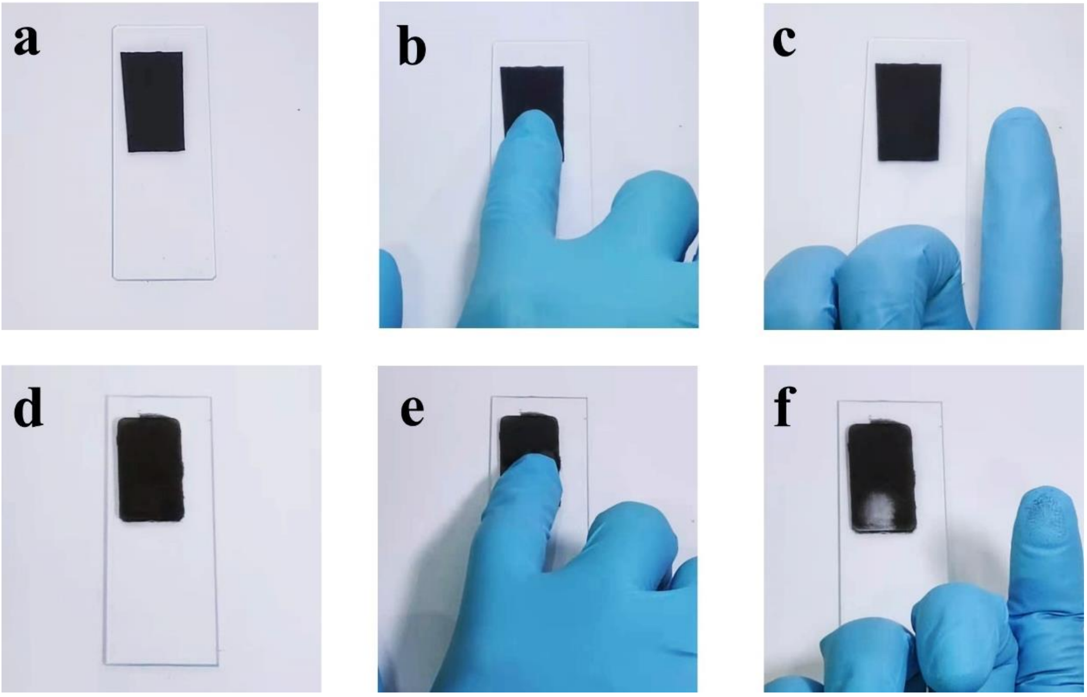

Supplementary Figure 3. The SCNs-PDMS films fabricated by different methods. (a) PVA sacrifice transfer method and (d) direct transfer to PDMS. (b/e) Rubbing the films with a finger. (c) The SCNs transferred to PVA sacrifice layer and then to PDMS was intact. (f) The SCNs transferred directly to PDMS substrate could be easily rubbed off.

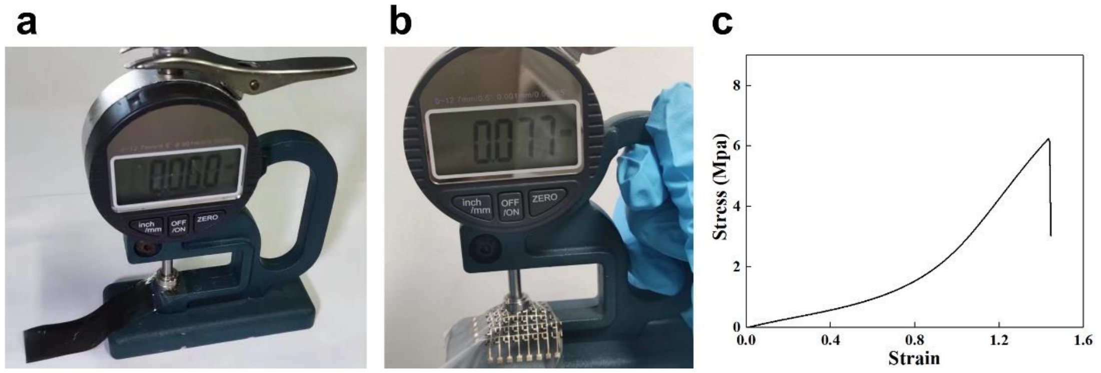

Supplementary Figure 4. The thickness of matrix; array film and tensile stress-strain curve of array film. (a) The thickness of PDMS layer. (b) The thickness of the sensor array. (c) The tensile stress-strain curve of the array film. Source data are provided as a Source Data file.

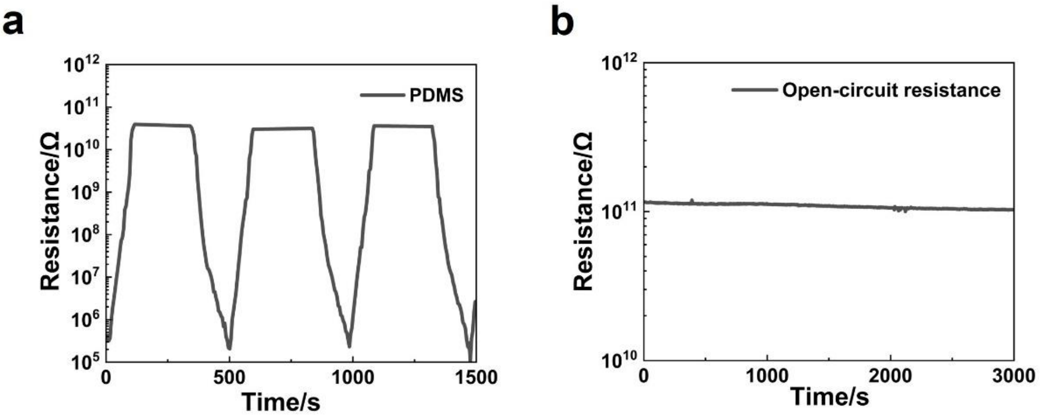

Supplementary Figure 5. The explanation data for the limitation of the sensing range. (a) 100% strain cyclic test of SCNs/PDMS (polydimethylsiloxane) sensor. (b) Open-circuit resistance of the high resistance meter. Source data are provided as a Source Data file.

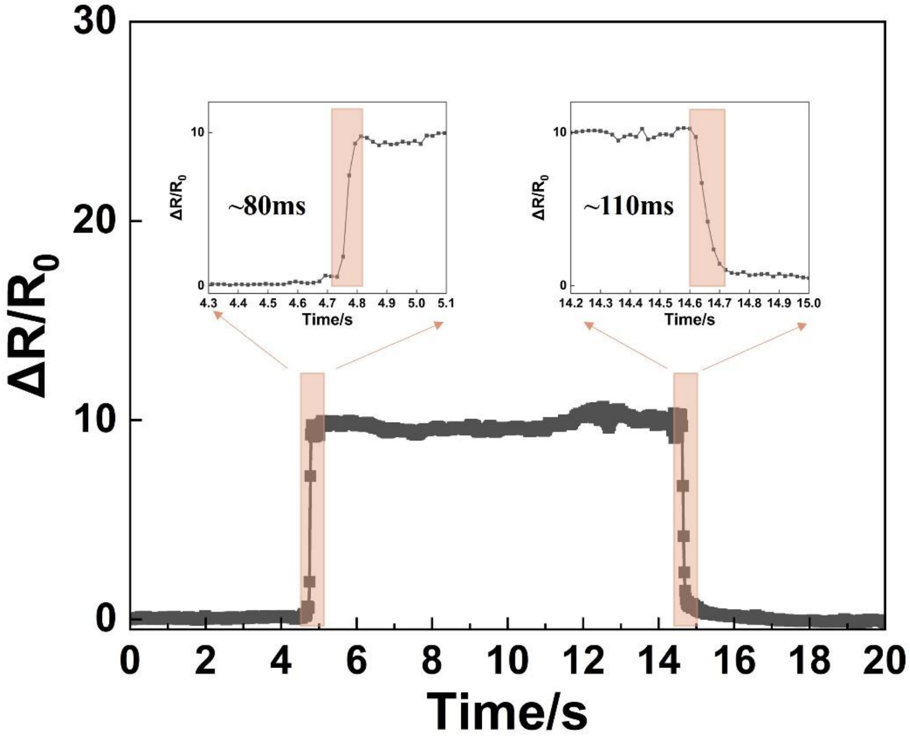

Supplementary Figure 6. Response time of SCNs/PDMS sensor. Source data are provided as a Source Data file.

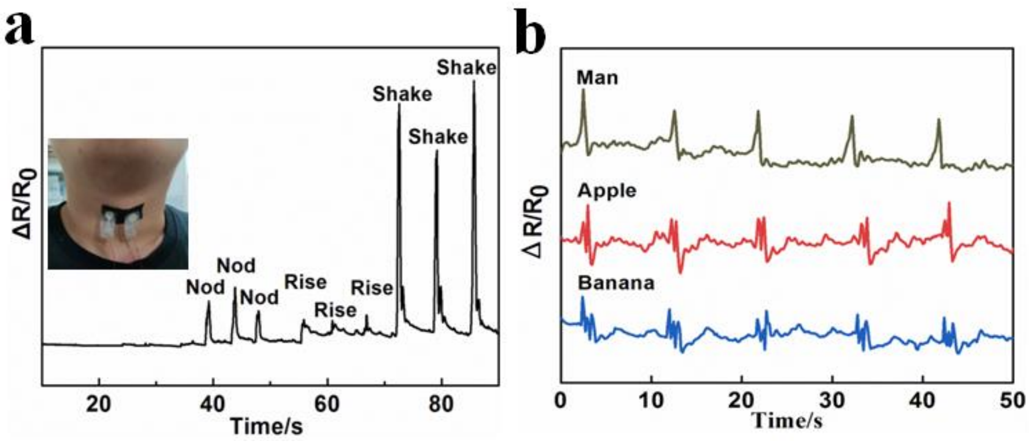

Supplementary Figure 7. Detecting human motions with the SCNs-based sensor. Normalized resistance changes ($\Delta R/R_0$) of the sensor when the volunteer made (a) large movements, such as nodding, raising or shaking his head; and (b) slight vibrations, such as pronouncing the words of "Man", "Apple", and "Banana". Source data are provided as a Source Data file.

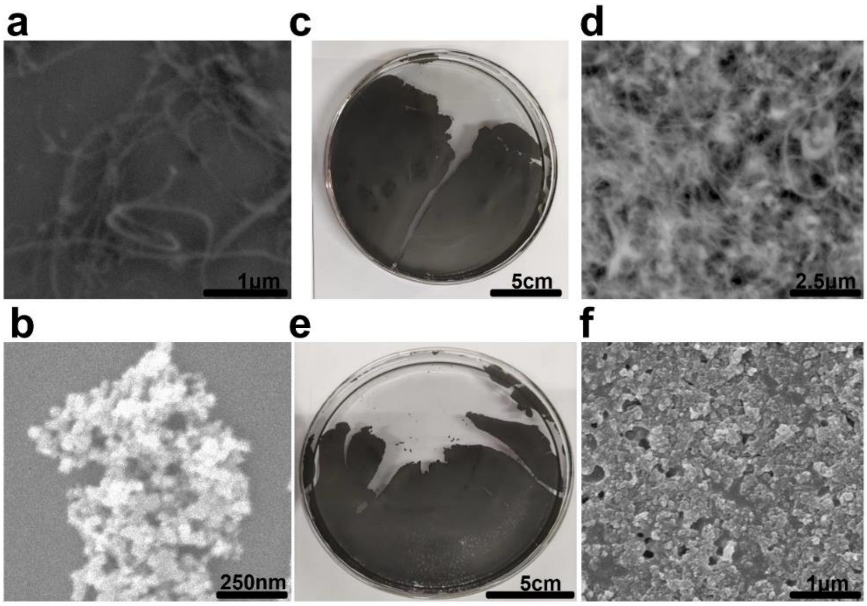

Supplementary Figure 8. Characterization of CB; CNT and their constructed films through self-assembly. SEM images of (a) CNT and (b) CB. The photograph and SEM images of the constructed films through self-assembly process with (c/d) CNT and (e/f) CB as conductive materials in the sensor.

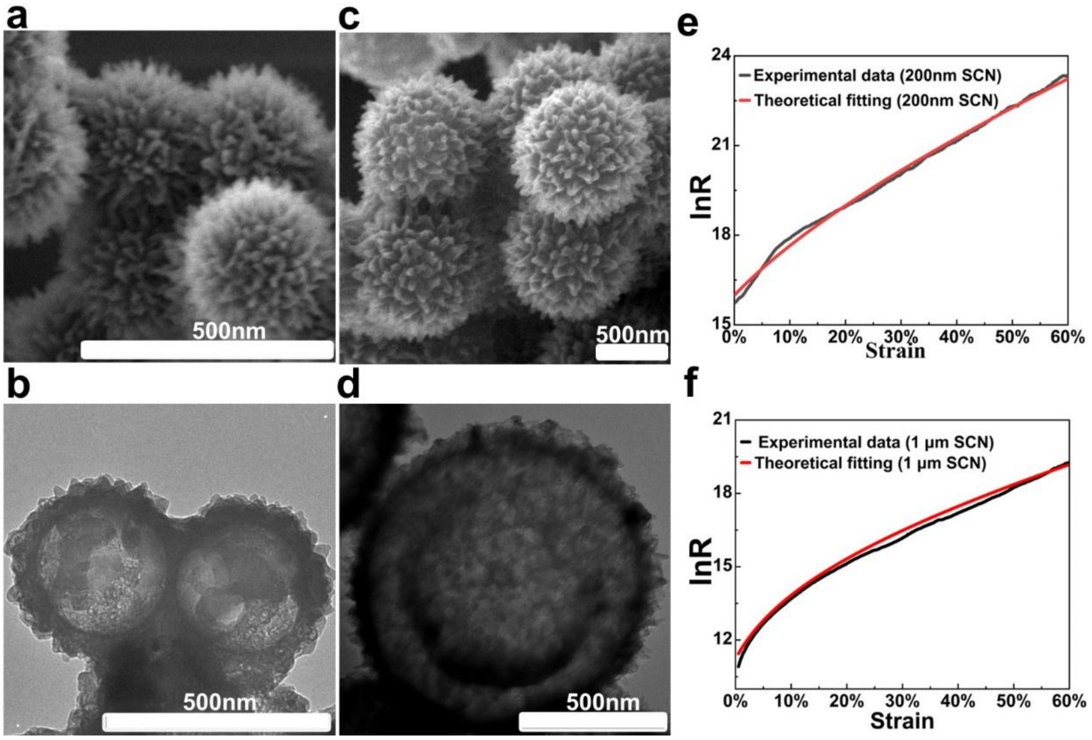

Supplementary Figure 9. Morphology and sensing behavior of SCNs with different diameters. (a) SEM and (b) TEM images of the SCNs (spike carbon nanospheres) with a diameter of 200 nm. (c) SEM and (d) TEM images of the SCNs with a diameter of 1μm. The theoretical fitting based on the proposed model corresponds well with the experimental data of the sensors fabricated with (e) 200 nm and (f) 1 μm of SCNs. Source data are provided as a Source Data file.

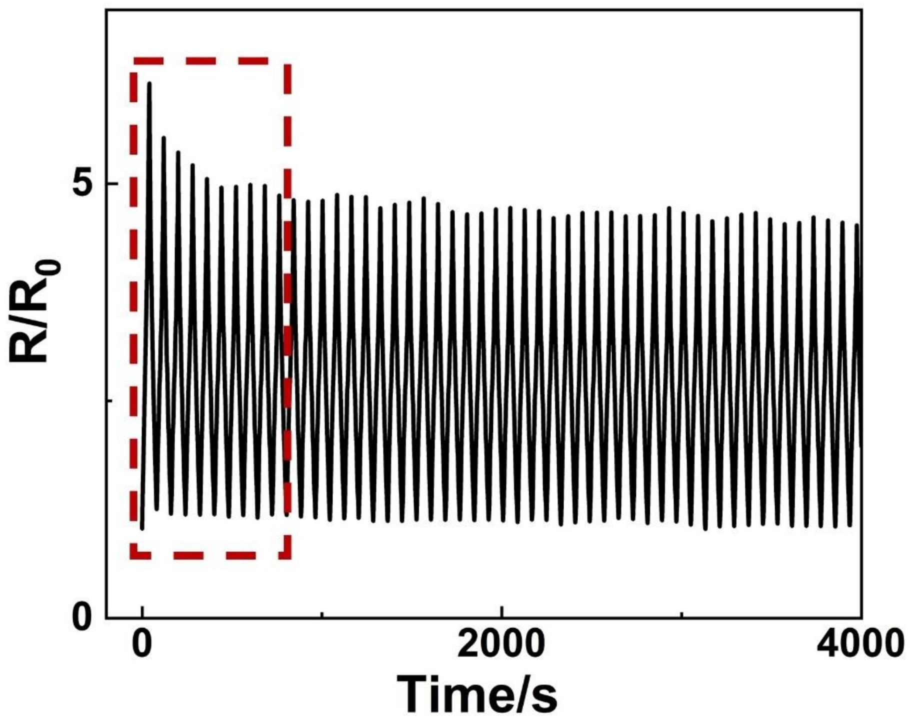

Supplementary Figure 10. The first 50 cycles from the 5000 cycles test in Figure 3f. Source data are provided as a Source Data file.

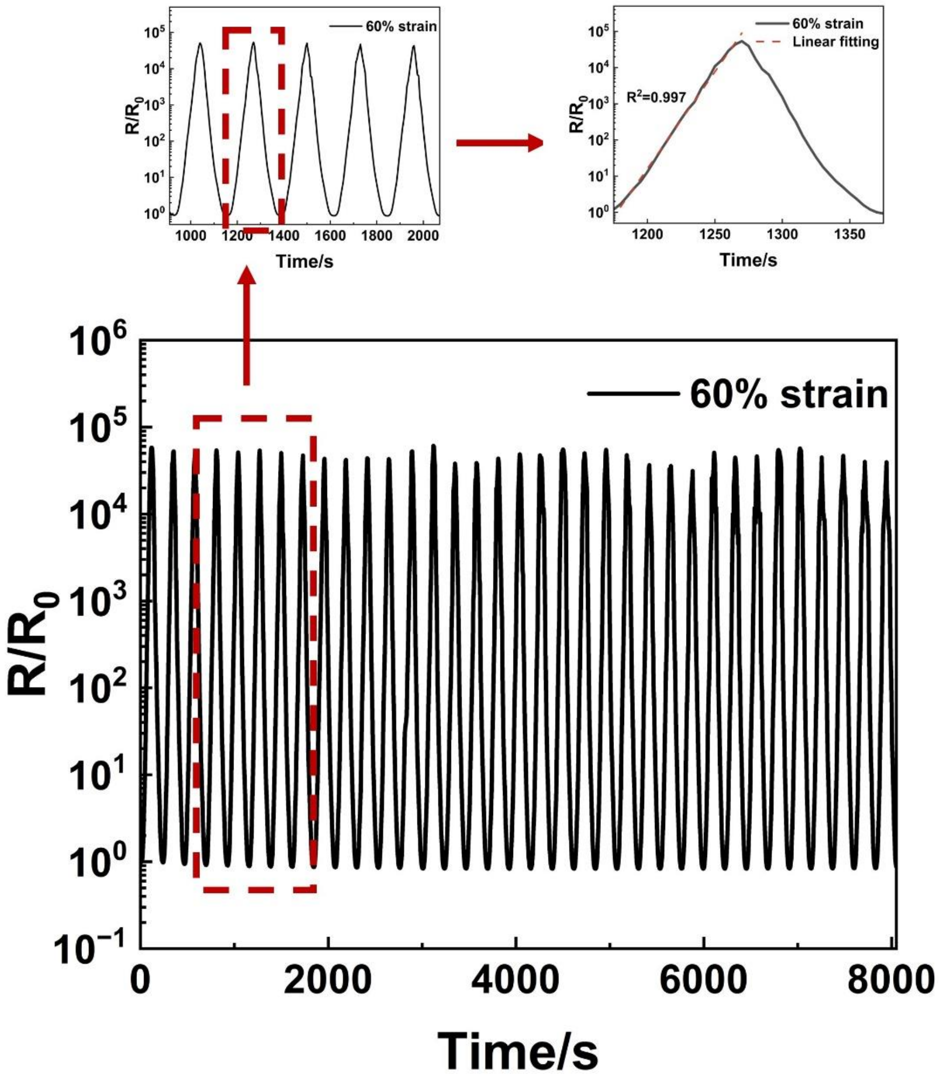

Supplementary Figure 11. 60% strain cyclic test on SCNs/PDMS sensor. Source data are provided as a Source Data file.

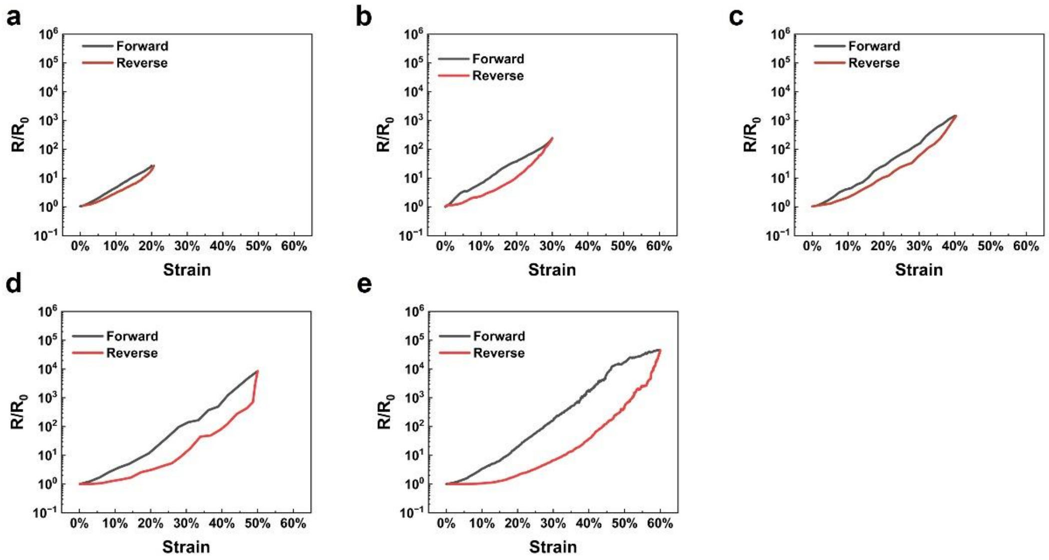

Supplementary Figure 12. Hysteresis tests of SCNs/PDMS sensor at different strains. (a) 20% strain; (b) 30% strain; (c) 40% strain; (d) 50% strain; (e) 60% strain. Source data are provided as a Source Data file.

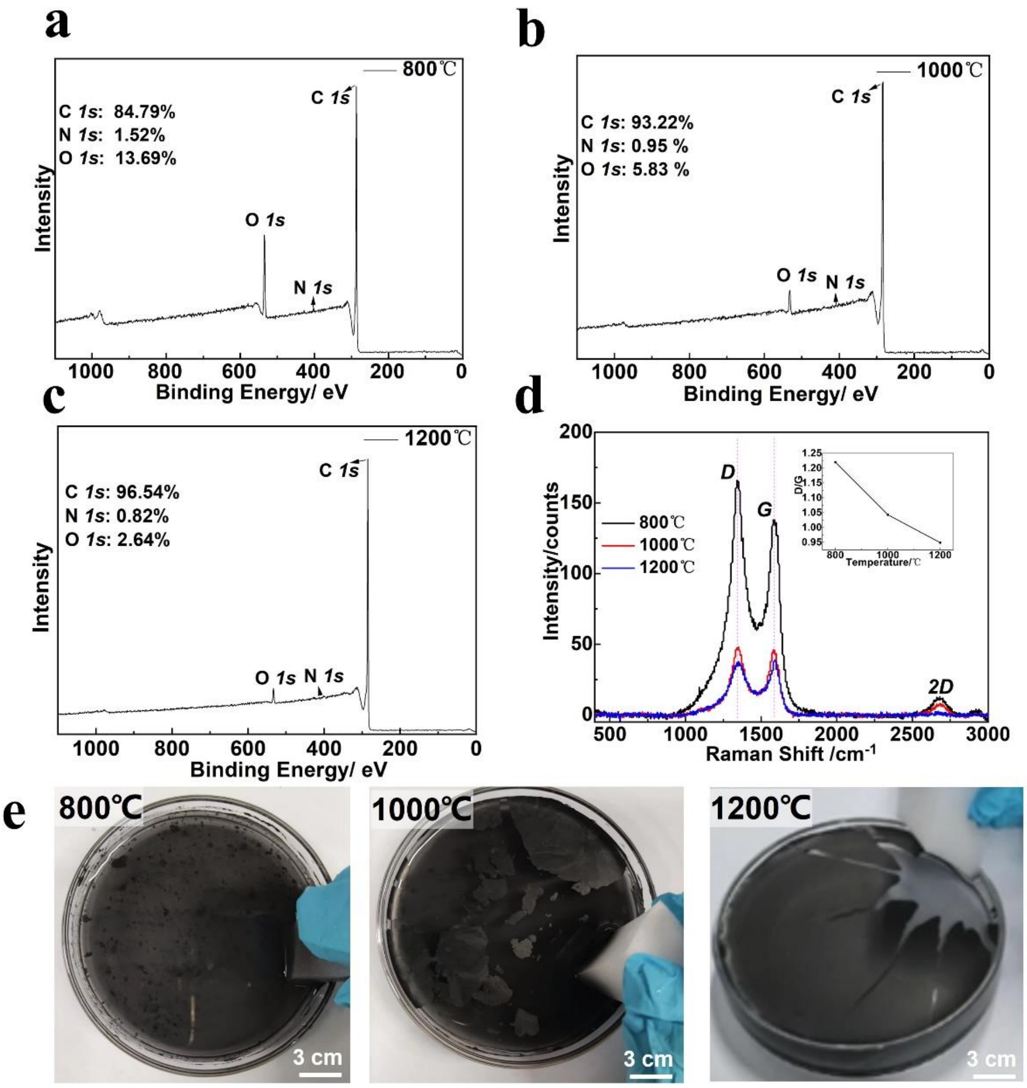

Supplementary Figure 13. Characterization of the carbonized SCNs. Temperature can effectively improve the SCNs hydrophobic properties. As the carbonization temperature increases from 800 ℃ to 1000℃ and 1200 ℃, oxygen contents of the SCNs from XPS characterizations were (a) 13.69%, (b) 5.83%, (c) 2.64%, respectively. (d) The *D/G* ratio in the Raman spectra also decreased from 1.22 to 0.949 when the temperature increases, indicating an increasing degree of graphitization of the SCNs. (e) Distributions of the SCNs after self-assemble process, where SCNs were carbonized at 800°C, 1000°C, 1200°C. Scale bar: 3 cm. Source data are provided as a Source Data file.

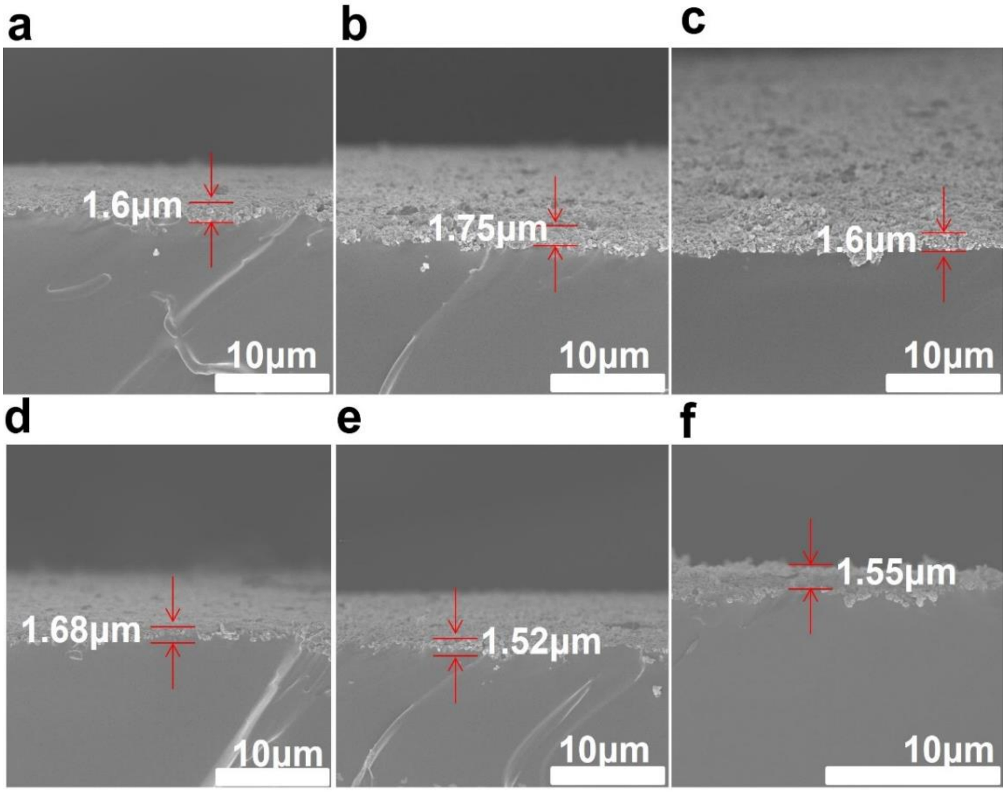

Supplementary Figure 14. Thickness of the SCN layers in the SCNs/PDMS composite films prepared with different concentrations of SCN. (a) C_{SCNs}=0.5 mg mL^{-1}; (b) C_{SCNs}=1.0 mg mL^{-1}; (c) C_{SCNs}=1.5 mg mL^{-1}; (d) C_{SCNs}=2.0 mg mL^{-1}; (e) C_{SCNs}=2.5 mg mL^{-1}; (f) C_{SCNs}=3.0 mg mL^{-1}.

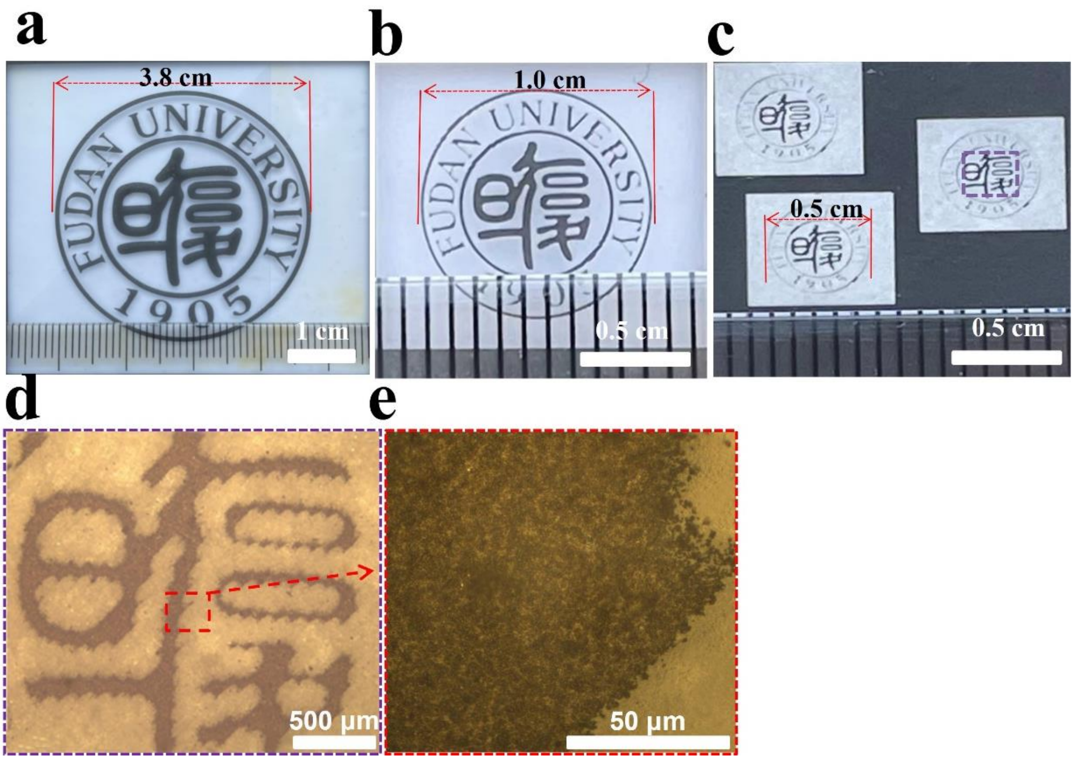

Supplementary Figure 15. Pictures of laser scribed logo. Logo of "Fudan" with diameters of (a) 3.8 cm, (b) 1.0 cm, (c) 0.5 cm, (d/e) magnified patterns with a diameter of 0.5 cm under optical microscope.

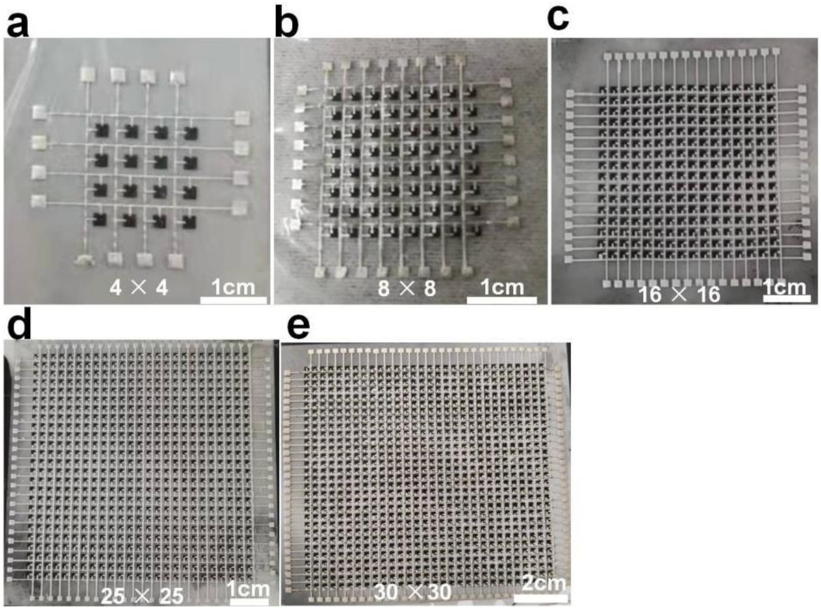

Supplementary Figure 16. The prepared sensor arrays with different scales and different sensing densities. (a) 4×4, 4 pixel cm^{-2}; (b) 8×8, 16 pixel cm^{-2}; (c) 16×16, 16 pixel cm^{-2}; (d) 25×25, 16 pixel cm^{-2}; (e) 30×30, 16 pixel cm^{-2}.

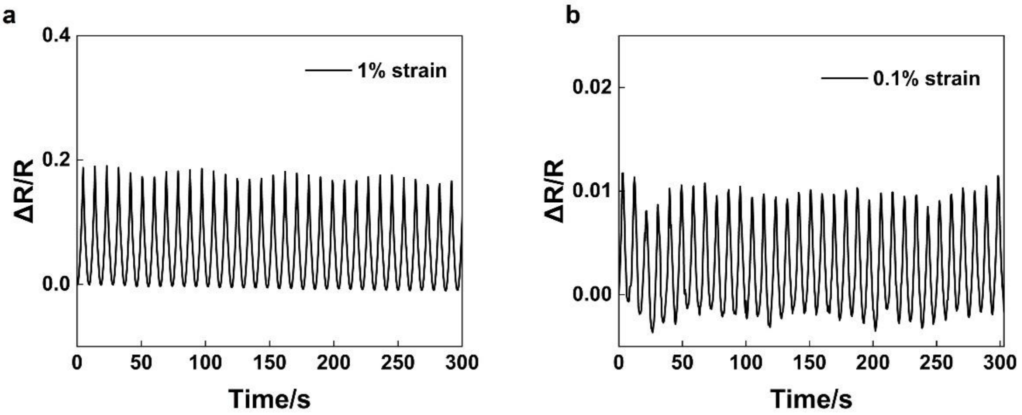

Supplementary Figure 17 The response of the SCNs/PDMS strain sensor to the cyclic tensile test. (a) 1% strain and (b) 0.1% strain. Source data are provided as a Source Data file.

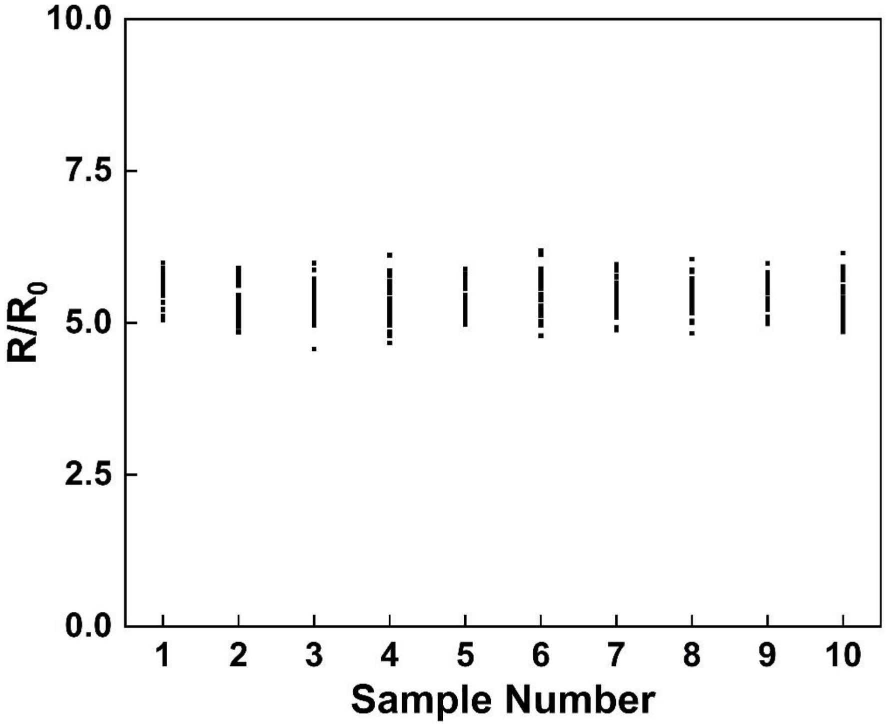

Supplementary Figure 18. Raw data of 10 sensor array films (30 × 30, 100 pixel/cm^2). The resistance changes from 36 randomly chosen sensing units from each film were presented in the figure under 10% uniform strain. Source data are provided as a Source Data file.

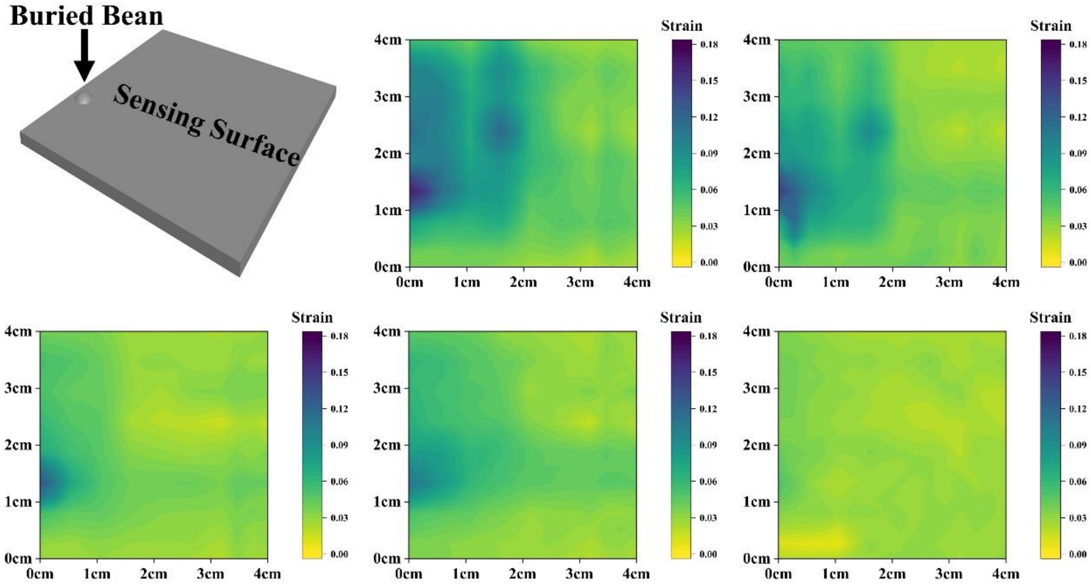

Supplementary Figure 19. Reconstructed strain field of the PDMS material with a mung bean at the bottom. Source data are provided as a Source Data file.

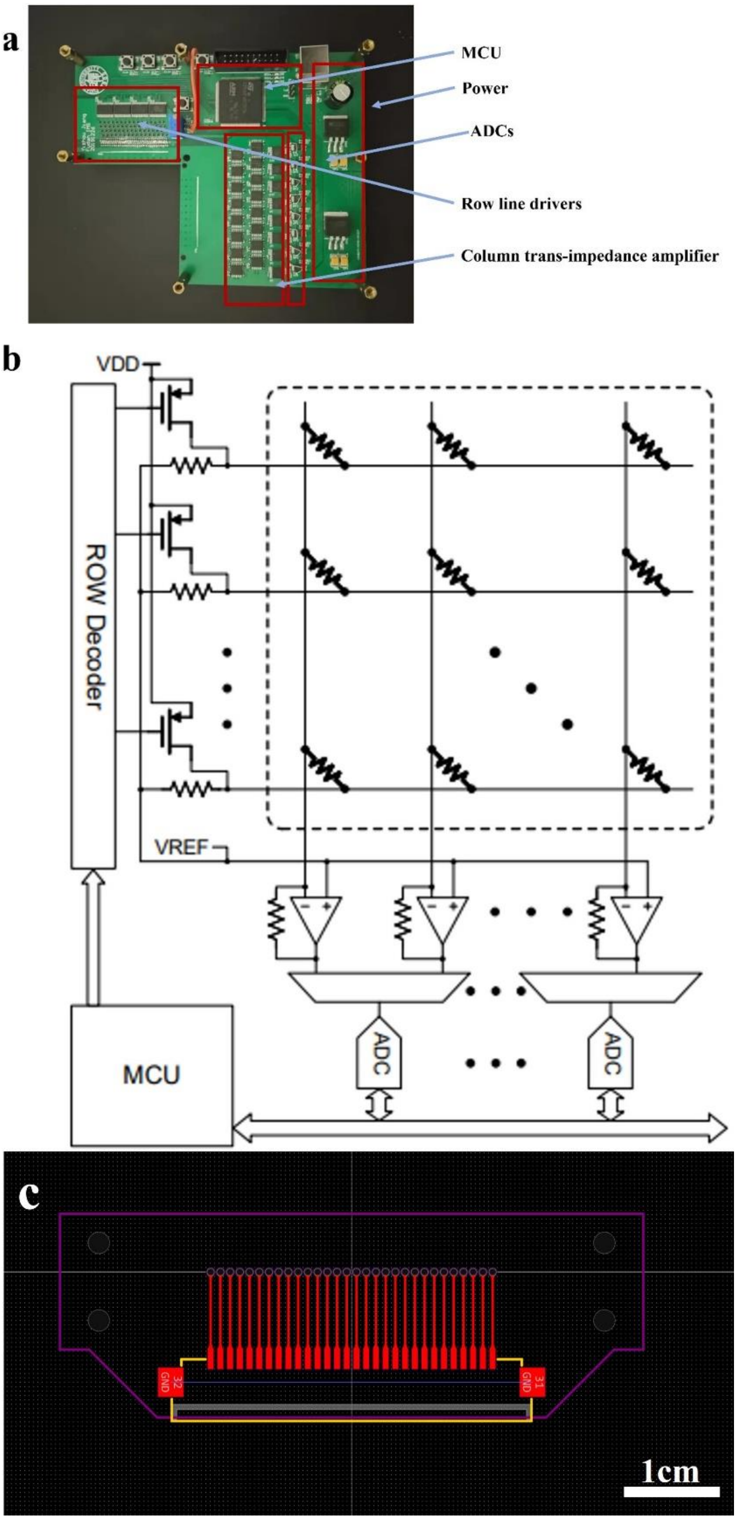

Supplementary Figure 20. Schematic diagrams of data acquisition system. (a) The component (MCU: microcontroller; ADC: analog-to-digital converters); (b) principal schematic of the system; (c) the adapter.

Supplementary Table 1. Performances of the reported strain sensing arrays

Materials	GF	Sensor Density /cm^{-2}	Strechability %	Sensing scales	Reference
SWCNTs-PDMS-PAA	2.0	1.25	100.0	1×5	2020[1]
AgNW/PDMS	130.0	0.58	10.0	4×9	2020[2]
AgNW-PDMS	20.0	2.56	4.0	4×4	2015[3]
In_2Se_3/PET-PDMS	237.0	30.0	0.4	5×5	2016[4]
Li/P_3HT-SBS	3.5	6.25	50	5×5	2022[5]
P_3HT-NFs/PDMS	32.0	0.70	100.0	19	2018[6]
Ag@CNT/PDMS	62.8	1.00	14.4	2×2	2020[7]
MWCNT/EA Resin	8.9	0.73	60.0	4×4	2021[8]
Carbon-based materials	3.3	0.54	0.1	25	2020[9]
Carbon paste	14.5	0.38	1.2	4×16	2022[10]
Ni-Au/PDMS	4500.0	0.11	1.4	1×4	2022[11]
GNP/PDMS	34.6	0.45	60.0	1×5	2022[12]
PANI-P(AAm-co-HEMA) hydrogels	11.0	4.00	300.0	6×8	2018[13]
Au-PDMS	350.0	2.00	80.0	4×5	2019[14]
GO-PDMS	2000.0	0.11	1.0	4×4	2021[15]
MCNT/CB-PDMS	8.0	1.00	35.0	1×3	2023[16]
SCNs-PDMS	70000.0	100.0	60.0	30×30	Our work

Supplementary Table 2. Performances of the stretchable strain sensors

Substrate	Conductive material	Stretch-ability/%	GF	Reference
PDMS	CNT/CB	35.0	8.0	2023[16]
Rubber substrate	MXene/CNT	130.0	772.6	2018[17]
Polyester	Ag fiber	50.0	140.0	2022[18]
PDMS	CNT/GNP	50.0	197.0	2021[19]
TPU	MWCNTs@MXene	100.0	363.0	2021[20]
Ecoflex	CNT	20.0	14.5	2021[21]
Dragon Skin	Graphene woven fabrics	274.0	2996.0	2018[22]
TPE	CNT	100.0	425.0	2018[23]
PU	GO–AgNW–C60	62.0	2392.9	2018[24]
Ecoflex	Carbonized silk fabrics	500.0	37.5	2016[25]
PDMS	Graphene oxide	200.0	1.5	2015[26]
PDMS	CNTs-CB	300.0	13.1	2018[27]
XSBR	CNT	217	25.98	2022[28]
Natural rubber	Graphene	110.0	139.0	2016[29]
PDMS	Ag nanowire	70	14.0	2014[30]
PDMS	MWCNT	30.0	62.9	2019[31]
Polystyrene-polyisoprene-polystyrene	MWCNT-GO	70.0	72.0	2017[32]
PDMS	Graphene	30.0	448.0	2019[33]
Cellulose	Mxene	10.0	399.5	2019[34]
TPU	Carbon fibers	5.0	85500.0	2020[35]
PDMS	Spiky-like carbon spheres	60.0	70000	This work

Supplementary Table 3. Statistical analysis of 10 sensor array films (30 × 30, 100 pixel/cm^2) after 10% uniform stretch

Sample number	Measured units	Average measured strain	Standard deviation	Standard error
1	36	10.26%	2.58%	
2	36	10.12%	3.33%	
3	36	10.02%	3.10%	
4	36	10.04%	3.77%	
5	36	10.05%	2.83%	
6	36	10.14%	3.82%	
7	36	10.09%	3.12%	
8	36	10.08%	2.95%	
9	36	10.14%	3.08%	
10	36	10.09%	3.27%	
				0.169%

Supplementary Notes 1: The derivation of λ

For an electrostatic field problem with no free electrons, the electric potential of the field follows Laplace equation:

$$\Delta u = 0 \tag{1}$$

where Δ is the Laplace operator, u is the electric potential. In spherical coordinates, it can be written as:

$$\frac{1}{r^2}\frac{\partial\left(r^2\frac{\partial u}{\partial r}\right)}{\partial r} + \frac{1}{r^2 sin\theta}\frac{\partial(sin\theta\frac{\partial u}{\partial\theta})}{\partial\theta} + \frac{1}{r^2 sin\theta^2}\frac{\partial^2\varphi}{\partial\varphi^2} = 0 \tag{2}$$

Using variable separation method towards to u:

$$u(r,\theta,\varphi) = R(r)Y(\theta,\varphi) \tag{3}$$

It is to be noted that the system is symmetric about the Z-axis, which means that φ is uncorrelated with $Y(\theta,\varphi)$. So, $u(r,\theta,\varphi)$ can be expressed as:

$$u(r,\theta) = R(r)Y(\theta) \tag{4}$$

So, the Laplace equation can be transformed into the equation below:

$$\frac{1}{R}\frac{\partial\left(r^2\frac{\partial R}{\partial r}\right)}{\partial r} = -\frac{1}{Y sin\theta}\frac{\partial(sin\theta\frac{\partial Y}{\partial\theta})}{\partial\theta} \tag{5}$$

The left formula is a function of r, while the right formula is a function of θ, so the two terms are equal only when they are both equal to a constant value. The constant here is recorded as $\lambda(\lambda+1)$ that can be determined by boundary condition, and we can get the following equations:

$$\frac{d\left(r^2\frac{dR}{dr}\right)}{dr} - \lambda(\lambda+1)R = 0 \tag{6}$$

$$\frac{1}{Y sin\theta}\frac{d(sin\theta\frac{dY}{d\theta})}{d\theta} + \lambda(\lambda+1) = 0 \tag{7}$$

The solution of (6) can be expressed as:

$$R = Ar^{\lambda} + B\frac{1}{r^{\lambda+1}} \tag{8}$$

Since $R(0)$ should be a limited value, $B = 0$.

In order to find the solution of (7), a change of variables is made here:

$$x = cos\theta \tag{9}$$

Substitute (9) into (8):

$$(1-x^2)\frac{d^2Y}{dx^2} - 2x\frac{dY}{dx} + \lambda(\lambda+1)Y = 0 \tag{10}$$

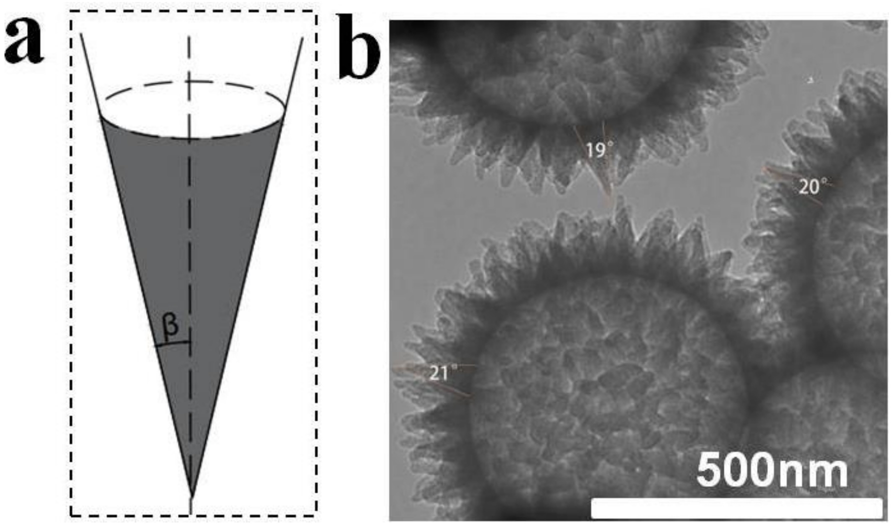

Supplementary Figure 21. Scheme of (a) half apex (β) in the spines, and (b) the actual apexes of the synthesized carbon sphere.

The boundary condition contains that $Y(cos\beta) = 0$ and $Y(1)$ is a limited value. So, the funtion $Y(x)$ can be expanded using a Taylor series expansion at $x = 1$

$$Y(x) = (1-x)^m \sum_{k=0}^{\infty} a_k (1-x)^k \tag{11}$$

Substitute (11) into (10), the coefficients of each power of $(1-x)$ should be zero, so:

$$\frac{a_{k+1}}{a_k} = \frac{(k-\lambda)(k+\lambda+1)}{2(k+1)^2} \tag{12}$$

$$m = 0 \tag{13}$$

Also, $Y(cos\beta) = 0$ gives a series of $\lambda_i (i = 1,2,3,\cdots)$, which constitute the sequence of eigenvalues.

$$u = \sum_{i=1}^{\infty} C_i\, r^{\lambda_i} Y_{\lambda_i}(cos\theta) \tag{14}$$

In order to approximates the electric potential near the origin of the coordinates, only the first term of (14) is retained.

$$u \approx Cr^{\lambda} Y_{\lambda}(cos\theta) \tag{15}$$

So, the electric potential u along z axis is proportional to r^{λ}, where λ is the smallest value of the series of λ_i which makes $Y(cos\beta) = 0$. For a conical tip conductor shape, $\beta \to 0$, and $\lambda \to 0$. Set $a_0 = 1$, with the help of Taylor series expansion equation $ln(1-x) = \sum_{i=1}^{\infty} -(\frac{x^i}{i})$, $Y_{\lambda}(cos\beta)$ can be expressed as:

$$Y_{\lambda}(cos\beta) \approx 1 - \lambda \sum_{i=1}^{\infty} \frac{[1 - sin^2(\frac{\beta}{2})]^k}{k} = 1 + \lambda \ln\left[sin^2\left(\frac{\beta}{2}\right)\right] = 0 \tag{16}$$

In our case, β is about 10° (Supplementary Figure 21-b), $sinx \approx x$, λ can be approximately calculated by:

$$\lambda \approx [2ln\left(\frac{2}{\beta}\right)]^{-1} \approx 0.2 \tag{17}$$

Supplementary Notes 2: Establishment of Sensing Mechanism Model

A model is proposed here to enhance the understanding of the sensitivity and linearity exhibited by the SCN-based sensors.

For SCNs in a conductive pathway, the potential difference between two spheres is assumed to be the same and we will get:

$$U_0 = \frac{U}{N-1} \tag{1}$$

where U is the external voltage, N is the number of SCNs in a conductive pathway, and U_0 represents the potential difference between two spheres. However, when considering the geometrical relationships between two adjacent spheres, the distance of the adjacent spheres can be calculated as:

$$d = \frac{L\sin\gamma}{N-1}\varepsilon + d_0 \tag{2}$$

where d is the distance of adjacent two spheres, ε is the strain applied on the sensor, L is length of the sensor, γ is the angle formed by the central axis of spines of spheres and the strain direction, and d_0 is the distance of two spheres before stretching. The parameters mentioned above are marked in Supplementary Figure 22. Here, we introduce L_0 and define it as:

$$L_0 = \frac{L\sin\gamma}{N-1} \tag{3}$$

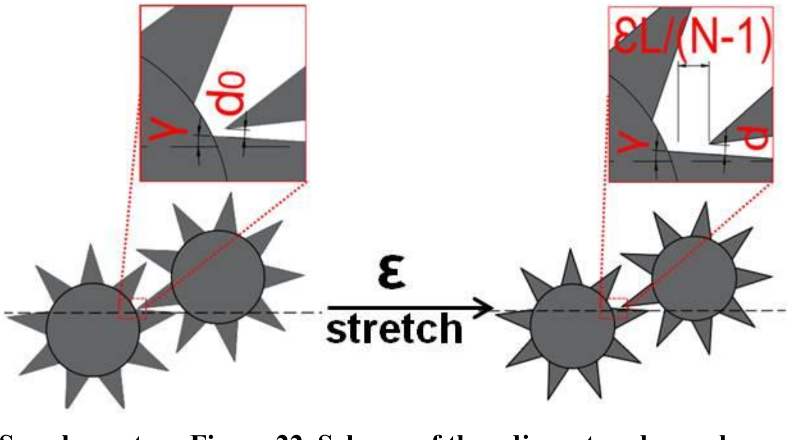

Supplementary Figure 22. Scheme of the adjacent carbon spheres.

In addition, taking the electric field concentration effect into consideration, the potential difference between two adjacent carbon spheres can also be expressed as (detailed derivation of λ has been discussed in Supplementary Notes 1):

$$U_0 = Cd^{\lambda} \tag{4}$$

where λ is concentration coefficient of the electric fields, and C is a constant. According to the equations of (1), (3) and (4), C can be expressed as:

$$C = \frac{U}{(N-1)(\varepsilon L_0 + d_0)^{\lambda}} \tag{5}$$

Then, the electric field strength can be expressed as:

$$E = \frac{\partial U_0}{\partial d} = \frac{U}{(N-1)(\varepsilon L_0 + d_0)^{\lambda}} \lambda d^{\lambda-1} \quad (6)$$

Substitute $d = \varepsilon L_0 + d_0$ into the above equation (6), the electric field strength of a spine in one sphere applied on surface of the other sphere can be expressed as:

$$E_d = \frac{U}{(N-1)} \lambda (\varepsilon L_0 + d_0)^{-1} \quad (7)$$

F-N equation gives the relationships between current density J and electric field strength E_d of two conduction layers sandwiched with an insulation layer:

$$J = AE_d^2 \exp\left(\frac{B}{E_d}\right) \quad (8)$$

where A and B are both constants, A is positive and B is negative. Substitute the E_d of equation (7) into the F-N equation, we can get the current density in one conductive path:

$$J = A(\frac{U\lambda}{N-1})^2 (\varepsilon L_0 + d_0)^{-2} \exp[\frac{B(N-1)}{U\lambda}(\varepsilon L_0 + d_0)] \quad (9)$$

Thus, the total current of all conductive paths can be expressed as:

$$I = \sum_{i=1}^{n} SA\left(\frac{U\lambda}{N_i - 1}\right)^2 (\varepsilon L_0 + d_0)^{-2} \exp\left[\frac{B(N_i - 1)}{U\lambda}(\varepsilon L_0 + d_0)\right] \quad (10)$$

Where N_i is the number for conductive path i, S is the area of a conductive path. n is the number of the conductive path. Note that the terms in (10) decrease with the increase of i, which means I is mainly determined by the first few terms. So, I can be approximately expressed as:

$$I \approx \sum_{i=1}^{m} SA\left(\frac{U\lambda}{N_i - 1}\right)^2 (\varepsilon L_0 + d_0)^{-2} \exp\left[\frac{B(N_i - 1)}{U\lambda}(\varepsilon L_0 + d_0)\right] \quad (11)$$

where m is the number of effective conductive pathway. Define N_0 as minimum number for all conductive path. Noticing that $N_0 \approx N_i$, $\frac{1}{N_i-1} \approx \frac{1}{N_0-1}$ for $i \in (0, m]$. I can be further approximately expressed as:

$$I \approx A\left(\frac{U\lambda}{N_0 - 1}\right)^2 (\varepsilon L_0 + d_0)^{-2} \exp\left[\frac{B(N_0 - 1)}{U\lambda}(\varepsilon L_0 + d_0)\right] \sum_{i=1}^{m} S \exp\left[\frac{B(N_i - N_0)}{U\lambda}(\varepsilon L_0 + d_0)\right] \quad (12)$$

Since $\frac{B(N_i-N_0)}{U\lambda}(\varepsilon L_0 + d_0) \to 0$, $\exp[\frac{B(N_i-N_0)}{U\lambda}(\varepsilon L_0 + d_0)] \approx \frac{B(N_i-N_0)}{U\lambda}(\varepsilon L_0 + d_0) + 1$. So, $\ln I$ can be approximately expressed as:

$$\ln I = \frac{B(N_0 - 1)L_0}{U\lambda}\varepsilon - 2\,ln\left(\varepsilon + \frac{d_0}{L_0}\right) + \ln\left\{\sum_{i=1}^{m} S[\frac{B(N_i - N_0)}{U\lambda}(\varepsilon L_0 + d_0) + 1]\right\} + \ln\left[\frac{A}{L_0^2}\left(\frac{U\lambda}{N_0 - 1}\right)^2\right] \quad (13)$$

Note that $\ln(x+1) \approx x$:

$$\ln\left\{\sum_{i=1}^{m} S[\frac{B(N_i - N_0)}{U\lambda}(\varepsilon L_0 + d_0) + 1]\right\} \approx \ln \mathrm{Sm} + \sum_{i=1}^{m} \frac{B(N_i - N_0)}{U\lambda m}(\varepsilon L_0 + d_0) \quad (14)$$

So, $\ln I$ can be further approximately expressed as:

$$\ln I \approx \frac{B(N_0 - 1)L_0}{U\lambda}\varepsilon - 2\ln\left(\varepsilon + \frac{d_0}{L_0}\right) + \ln \mathrm{Sm} + \sum_{i=1}^{m} \frac{B(N_i - N_0)}{U\lambda m}(\varepsilon L_0 + d_0) + \ln\left[\frac{A}{L_0^2}\left(\frac{U\lambda}{N_0 - 1}\right)^2\right] \quad (15)$$

After merging similar terms and we will get:

$$\ln I = \frac{B[N_0 - 1 + \sum_{i=1}^{m} \frac{(N_i - N_0)}{m}]L_0}{U\lambda}\varepsilon - 2\ln\left(\varepsilon + \frac{d_0}{L_0}\right) + \sum_{i=1}^{m} \frac{B(N_i - N_0)d_0}{U\lambda m} + \ln\left[\frac{ASm}{L_0^2}\left(\frac{U\lambda}{N_0 - 1}\right)^2\right] \quad (16)$$

Thus, $D = \sum_{i=1}^{m} \frac{(N_i - N_0)}{m}$ and $F = \sum_{i=1}^{m} \frac{B(N_i - N_0)d_0}{U\lambda m} + \ln\left[\frac{ASm}{L_0^2}(\frac{U\lambda}{N_0 - 1})^2\right]$. So, the apparent ohmic resistance R can be expressed as:

$$\ln R = -\frac{B(N_0 - 1 + D)L_0}{U\lambda}\varepsilon + 2\ln\left(\varepsilon + \frac{d_0}{L_0}\right) + \ln \mathrm{U} - F \quad (17)$$

The sensitivity of the sensor is proportional to $\frac{1}{\lambda}$, which is positively related to sharpness of the spines. Besides, equation (3) indicates that increase of the distance between two adjacent spheres is much smaller compared to the increase of their relative displacement due to geometric effect of the spines. This may be an explanation of how tunneling effect can be maintained during a large range of 0-60% stretch. The $R{\sim}\varepsilon$ relationship of our sensor unit can be simplified according to the data from Figure 3e as:

$$\ln R = (16.739 \pm 0.029)\,\varepsilon + 12.036 \pm 0.010 \quad (18)$$

Supplementary Notes 3: the explanation of the minimum value of $\frac{d_0}{L_0}$

For closely packed spheres, d_0 is ought to be close to zero. However, since the device is functional, and the tunneling effect do exist, the average electric field strength between two spheres should be smaller than the breakdown electric field strength:

$$\bar{E} < E_{break} \quad (1)$$

Practically, E is a concave function, thus, $\bar{E} > E(\frac{d_{min}}{2})$

$$\frac{2U}{(N-1)}\lambda(d_{min})^{-1} < E_{break} \quad (2)$$

where E_{break} is the breakdown electric field strength for air. Meanwhile, L_0 is defined to be:

$$L_0 = \frac{L\sin\gamma}{N-1} \quad (3)$$

Combining (1) and (2), we can get the minimum value of $\frac{d_0}{L_0}$

$$\frac{d_0}{L_0} > \frac{2U\lambda}{L\sin\gamma E_{break}} \quad (4)$$

In our case, $U = 40\ V$, $\lambda = 0.2$, $\gamma \approx 10°$, $L = 1.5 \times 10^{-3}\ m$. $E_{break} \approx 4.5 \times 10^5\ V\ m^{-1}$ for air in uneven electric field[36].

$$\frac{d_0}{L_0} > 0.138 \quad (5)$$

Supplementary Notes 4: Mechanical Analysis of Floating SCN

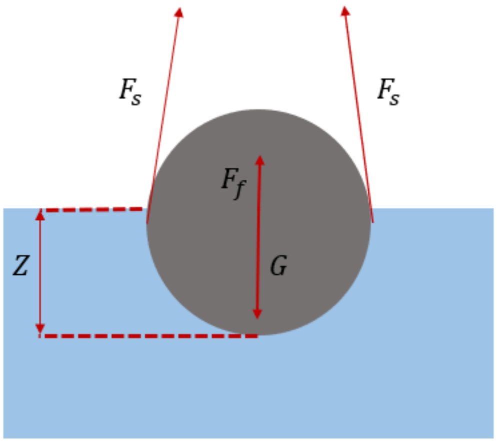

Supplementary Figure 23. Scheme of a nanosphere floats on the water.

When a nanosphere floats on the water, it will be subject to gravity (G), buoyancy (F_f) and surface tension (F_s). The forces can be expressed as:

$$G = mg \tag{1}$$

$$F_f = \pi\rho g\left(RZ^2 - \frac{Z^3}{3}\right) \tag{2}$$

$$F_s = 2\pi\sigma\sqrt{2RZ - Z^2} \tag{3}$$

ρ is density of the water, R is radius of the sphere, Z is the depth of sphere in water, σ is the coefficient of the surface tension. The resultant upward force $F_r(Z)$ consists of F_f and F_s, and it can be expressed as:

$$F_r(Z) = \pi\rho g\left(RZ^2 - \frac{Z^3}{3}\right) + 2\pi\sigma\sqrt{2RZ - Z^2} \tag{4}$$

For an equilibrium system, $G = F_f + F_s$. Maximum value of $F_r(Z)$ equals to the maximum mass a sphere can afford without sinking. In order to obtain the maximum value of $F_r(Z)$. The derivative of $F_r(Z)$ is calculated and set to be zero:

$$\frac{dF_r}{dZ} = \pi\rho g(2RZ - Z^2) + 2\pi\sigma\frac{R - Z}{\sqrt{2RZ - Z^2}} = 0 \tag{5}$$

Substitute $R - Z$ to M, ($M = R - Z$):

$$2\sigma\frac{M}{\sqrt{(R - M)(R + M)}} = -\rho g(R - M)(R + M) \tag{6}$$

Noticing that $M < 0$, square both sides of this equation and we will get:

$$4\left(\frac{\sigma M}{\rho g}\right)^2 = (R^2 - M^2)^3 \tag{7}$$

Substitute $R^2 - M^2$ to t, ($t = R^2 - M^2$), and set $a = \left(\frac{2\sigma}{\rho g}\right)^2$:

$$t^3 + at - aR^2 = 0 \quad (8)$$

$$t = \sqrt[3]{\frac{aR^2}{2} + \sqrt{(\frac{aR^2}{2})^2 + (\frac{a}{3})^3}} + \sqrt[3]{\frac{aR^2}{2} - \sqrt{(\frac{aR^2}{2})^2 + (\frac{a}{3})^3}} \quad (9)$$

$$Z_0 = R + \sqrt{R^2 - t} \quad (10)$$

$$F_{rmax}(Z) = F_r(Z_0) = m_{max}g \quad (11)$$

In a LB film, every sphere in the bottom layer gives a maximum resultant upward force $\mathrm{F_{rmax}}(Z)$. Since $\mathrm{F_{rmax}}(Z)$ depends only on the coefficient of surface tension and the radius of the sphere and can be treated as a constant, the number of spheres that the bottom spheres can sustain is a constant in our case. If m_{max} is smaller than the mass of a single sphere, the formation of a continuous film is infeasible. Even if m_{max} exceeds that of one sphere, maximum thickness of LB film has no correlation with the additional added spheres. The results agree with that of SEM observations (Supplementary Figure 14).

Reference

1. Wang, M. *et al.* Gesture recognition using a bioinspired learning architecture that integrates visual data with somatosensory data from stretchable sensors. *Nat. Electron.* **3**, 563-570 (2020).
2. Li, M., Chen, S., Fan, B., Wu, B. & Guo, X. Printed flexible strain sensor array for bendable interactive surface. *Adv. Funct. Mater.* **30**, 2003214 (2020).
3. Kim, K.K. *et al.* Highly sensitive and stretchable multidimensional strain sensor with prestrained anisotropic metal nanowire percolation networks. *Nano Lett.* **15**, 5240-5247 (2015).
4. Feng, W. *et al.* Sensitive electronic-skin strain sensor array based on the patterned two-dimensional α-In_2Se_3. *Chem. Mater.* **28**, 4278-4283 (2016).
5. Son, H.J., Jeong, S., Jeong, I., Kim, H.-J. & Park, M. Stretchable and conductive Li-complexed poly(3-hexylthiophene) nanofibrils/elastomer composites for printed electronic skins. *ACS Appl. Nano Mater.* **5**, 13027-13036 (2022).
6. Kim, H.-J., Thukral, A. & Yu, C. Highly sensitive and very stretchable strain sensor based on a rubbery semiconductor. *ACS Appl. Mater. Interfaces.* **10**, 5000-5006 (2018).
7. Pei, Z. *et al.* A high gauge-factor wearable strain sensor array via 3D printed mold fabrication and size optimize of silver coated carbon nanotubes. *Nanotechnology* **31**, 305501 (2020).
8. Xiao, T. *et al.* 3d printing of flexible strain sensor array based on uv-curable multiwalled carbon nanotube/elastomer composite. *Adv. Mater. Technol.* **6**, 2000745 (2021).
9. Zymelka, D., Togashi, K. & Kobayashi, T. Carbon-based printed strain sensor array for remote and automated structural health monitoring. *Smart Mater. Struct.* **29**, 105022 (2020).
10. Yu, P. *et al.* Arbitrary-shape-adaptable strain sensor array with optimized circuit layout via direct-ink-writing: Scalable design and hierarchical printing. *Mater. Des.* **214**, 110388 (2022).
11. Wan, Y. *et al.* Flexible intelligent sensing system for plane complex strain monitoring. *Adv. Mater. Technol.* **7**, 2200386 (2022).
12. Wang, G., Wang, M., Zheng, M., Yao, S. & Ebo, B. High-sensitivity GNPS/PDMS flexible strain sensor with a microdome array. *ACS Appl. Electron. Mater.* **4**, 4576 (2022).
13. Wang, Z. *et al.* Ultrastretchable strain sensors and arrays with high sensitivity and linearity based on super tough conductive hydrogels. *Chem. Mater.* **30**, 8062-8069 (2018).

14. Shi, J. *et al.* Crack control in biotemplated gold films for wide-range, highly sensitive strain sensing. *ACS Appl. Mater. Interfaces* **6**, 1901223 (2019).

15. Xu, W. *et al.* Patterning of graphene for highly sensitive strain sensing on various curved surfaces. *Nano Sel.* **2**, 121-128 (2021).

16. Lee, J.H. *et al.* Heterogeneous structure omnidirectional strain sensor arrays with cognitively learned neural networks. *Adv. Mater.* **35**, 2208184 (2023).

17. Cai, Y. *et al.* Stretchable Ti_3C2Tx MXene/carbon nanotube composite based strain sensor with ultrahigh sensitivity and tunable sensing range. *ACS Nano* **12**, 56-62 (2018).

18. Li, J., Li, S. & Su, Y. Stretchable strain sensors based on deterministic-contact-resistance braided structures with high performance and capability of continuous production. *Adv. Funct. Mater.* **32**, 2208216 (2022).

19. Li, Y. *et al.* Hybrid strategy of graphene/carbon nanotube hierarchical networks for highly sensitive, flexible wearable strain sensors. *Sci. Rep.* **11**, 21006 (2021).

20. Wang, H. *et al.* High-performance foam-shaped strain sensor based on carbon nanotubes and $Ti_3C_2T_x$ MXene for the monitoring of human activities. *ACS Nano* **15**, 9690-9700 (2021).

21. Qaiser, N. *et al.* A robust wearable point-of-care CNT-based strain sensor for wirelessly monitoring throat-related illnesses. *Adv. Funct. Mater.* **31**, 2103375 (2021).

22. He, T., Lin, C., Shi, L., Wang, R. & Sun, J. Through-layer buckle wavelength-gradient design for the coupling of high sensitivity and stretchability in a single strain sensor. *ACS Appl. Mater. Interfaces* **10**, 9653-9662 (2018).

23. Zhou, J., Xu, X., Xin, Y. & Lubineau, G. Coaxial thermoplastic elastomer-wrapped carbon nanotube fibers for deformable and wearable strain sensors. *Adv. Funct. Mater.* **28**, 1705591 (2018).

24. Shi, X., Liu, S., Sun, Y., Liang, J. & Chen, Y. Lowering internal friction of 0D–1D–2D ternary nanocomposite-based strain sensor by fullerene to boost the sensing performance. *Adv. Funct. Mater.* **28**, 1800850 (2018).

25. Wang, C. *et al.* Carbonized silk fabric for ultrastretchable, highly sensitive, and wearable strain sensors. *Adv. Mater.* **28**, 6640-6648 (2016).

26. Cheng, Y., Wang, R., Sun, J. & Gao, L. A stretchable and highly sensitive graphene-based fiber for sensing tensile strain, bending, and torsion. *Adv. Mater.* **27**, 7365-7371 (2015).

27. Zheng, Y. *et al.* A highly stretchable and stable strain sensor based on hybrid carbon nanofillers/polydimethylsiloxane conductive composites for large human motions monitoring. *Compos. Sci. Technol.* **156**, 276-286 (2018).

28. Lin, M. *et al.* A high-performance, sensitive, wearable multifunctional sensor based on rubber/CNT for human motion and skin temperature detection. *Adv. Mater.* **34**, 2107309 (2022).

29. Lin, Y. *et al.* Graphene–elastomer composites with segregated nanostructured network for liquid and strain sensing application. *ACS Appl. Mater. Interfaces* **8**, 24143-24151 (2016).

30. Amjadi, M., Pichitpajongkit, A., Lee, S., Ryu, S. & Park, I. Highly stretchable and sensitive strain sensor based on silver nanowire elastomer nanocomposite. *ACS Nano*, **8**, 5154–5163 (2014).

31. Chen, Y.-F. *et al.* Achieving highly electrical conductivity and piezoresistive sensitivity in polydimethylsiloxane/multi-walled carbon nanotube composites via the incorporation of silicon dioxide micro-particles. *Compos. Sci. Technol.*, **177**, 41-48 (2019).

32. Kim, J.Y. *et al.* 3D printable composite dough for stretchable, ultrasensitive and body-patchable strain sensors. *Nanoscale* **9**, 11035-11046 (2017).

33. Huang, K. *et al.* Three-dimensional printing of a tunable graphene-based elastomer for strain sensors with ultrahigh sensitivity. *Carbon* **143**, 63-72 (2019).

34. Cao, W.-T. *et al.* Mxene-reinforced cellulose nanofibril inks for 3D-printed smart fibres and textiles. *Adv. Funct. Mater.* **29**, 1905898 (2019).

35. Araromi, O.A. *et al.* Ultra-sensitive and resilient compliant strain gauges for soft machines. *Nature* **587**, 219-224 (2020).

36. Allen, N.L., Boutlendj, M., Hughes, R.C., Lightfoot, H.A. & Neville, R.B. Analysis and comparison of rod plane and rod rod gaps as direct voltage measurement devices. *IEE Proc. A. Sci. Meas. Technol.* **139**, 279-284 (1992).
