# Supplementary material for: High-density, highly sensitive sensor array of spiky carbon nanospheres for strain field mapping
Source: Nat Commun. 2024 May 4;15:3752. doi: 10.1038/s41467-024-47283-8 (PMC11069524; doi:10.1038/s41467-024-47283-8)
Supplement: Supplementary file 3 — Description of additional supplementary files [file 41467_2024_47283_MOESM3_ESM.pdf]

DESCRIPTION OF ADDITIONAL SUPPLEMENTARY FILES

Supplementary Movie 1: The formation of closely packed SCNs film on the water surface.

Supplementary Movie 2: Process of achieving electrical interconnection for a sensor array.

Supplementary Movie 3: Real-time strain detection of sensing film when attached to a hardwood board.

Supplementary Movie 4: Real-time strain detection of sensing film when attached to a soft sponge.

Supplementary Movie 5: Illustration of the process for building a pork strain monitoring platform.

Supplementary Movie 6: Real-time strain detection of sensing film when attached to a pork slice.

Supplementary Movie 7: The process of laser scribing for sensor array patterning
