# Supplementary material for: High-density, highly sensitive sensor array of spiky carbon nanospheres for strain field mapping
Source: Nat Commun. 2024 May 4;15:3752. doi: 10.1038/s41467-024-47283-8 (PMC11069524; doi:10.1038/s41467-024-47283-8)
Supplement: Supplementary file 11 — Reporting Summary [file 41467_2024_47283_MOESM11_ESM.pdf]

Corresponding author(s): Limin Wu; Zhuo Li

Last updated by author(s): Jan 20, 2024

Reporting Summary

Nature Portfolio wishes to improve the reproducibility of the work that we publish. This form provides structure for consistency and transparency in reporting. For further information on Nature Portfolio policies, see our Editorial Policies and the Editorial Policy Checklist.

Statistics

For all statistical analyses, confirm that the following items are present in the figure legend, table legend, main text, or Methods section.

n/a	Confirmed	
☐	☒	The exact sample size (*n*) for each experimental group/condition, given as a discrete number and unit of measurement
☐	☒	A statement on whether measurements were taken from distinct samples or whether the same sample was measured repeatedly
☒	☐	The statistical test(s) used AND whether they are one- or two-sided<br>*Only common tests should be described solely by name; describe more complex techniques in the Methods section.*
☒	☐	A description of all covariates tested
☒	☐	A description of any assumptions or corrections, such as tests of normality and adjustment for multiple comparisons
☐	☒	A full description of the statistical parameters including central tendency (e.g. means) or other basic estimates (e.g. regression coefficient) AND variation (e.g. standard deviation) or associated estimates of uncertainty (e.g. confidence intervals)
☒	☐	For null hypothesis testing, the test statistic (e.g. *F*, *t*, *r*) with confidence intervals, effect sizes, degrees of freedom and *P* value noted<br>*Give P values as exact values whenever suitable.*
☒	☐	For Bayesian analysis, information on the choice of priors and Markov chain Monte Carlo settings
☒	☐	For hierarchical and complex designs, identification of the appropriate level for tests and full reporting of outcomes
☒	☐	Estimates of effect sizes (e.g. Cohen's *d*, Pearson's *r*), indicating how they were calculated

Our web collection on statistics for biologists contains articles on many of the points above.

Software and code

Policy information about availability of computer code

Data collection	KickStart 2.6.0 (High resistivity measurement module)
Data analysis	Origin 9 and origin 2023b were used for plotting and data analysis. Python 3.9 (pyqtgraph module) was used for real-time data analysis and plotting.

For manuscripts utilizing custom algorithms or software that are central to the research but not yet described in published literature, software must be made available to editors and reviewers. We strongly encourage code deposition in a community repository (e.g. GitHub). See the Nature Portfolio guidelines for submitting code & software for further information.

Data

Policy information about availability of data

All manuscripts must include a data availability statement. This statement should provide the following information, where applicable:

- Accession codes, unique identifiers, or web links for publicly available datasets
- A description of any restrictions on data availability
- For clinical datasets or third party data, please ensure that the statement adheres to our policy

The data supporting the conclusion of this study have been deposited in “figshare” at https://doi.org/10.6084/m9.figshare.25020161. Additional data related to the article may be requested from the author.

Research involving human participants, their data, or biological material

Policy information about studies with human participants or human data. See also policy information about sex, gender (identity/presentation), and sexual orientation and race, ethnicity and racism.

Reporting on sex and gender	No experiment or statement is related to sex or gender in this manuscript.
Reporting on race, ethnicity, or other socially relevant groupings	No socially constructed or socially relevant categorization variables are relevant in manuscript.
Population characteristics	No human participants or human data is relevant in manuscript.
Recruitment	No participant was recruited in manuscript.
Ethics oversight	Fudan University

Note that full information on the approval of the study protocol must also be provided in the manuscript.

Field-specific reporting

Please select the one below that is the best fit for your research. If you are not sure, read the appropriate sections before making your selection.

☒ Life sciences ☐ Behavioural & social sciences ☐ Ecological, evolutionary & environmental sciences

For a reference copy of the document with all sections, see nature.com/documents/nr-reporting-summary-flat.pdf

Life sciences study design

All studies must disclose on these points even when the disclosure is negative.

Sample size	We have prepared at least 5 or more parallel samples for every related experiment. Given that our experiment is not highly complex and the experimental data shows relatively low variations, we believe that our experimental size is appropriate.
Data exclusions	No data was excluded.
Replication	Repetitive tests for sample preparation and sensing signal has been conducted to demonstrate the good reproducibility of the experiment. The specific data is presented in the manuscript.
Randomization	Our experiment only involves testing the electrical performance of samples and did not involve allocation issues. The covariates that may affected this experiment mainly include air humidity, temperature, etc. In our laboratory testing environment, the variations of these covariates were negligible.
Blinding	Considering that our experiment does not involve human or other biological participation, we believe that "blinding" is not relevant to our study.

Reporting for specific materials, systems and methods

We require information from authors about some types of materials, experimental systems and methods used in many studies. Here, indicate whether each material, system or method listed is relevant to your study. If you are not sure if a list item applies to your research, read the appropriate section before selecting a response.

Materials & experimental systems

n/a	Involved in the study	
☒	☐	Antibodies
☒	☐	Eukaryotic cell lines
☒	☐	Palaeontology and archaeology
☒	☐	Animals and other organisms
☒	☐	Clinical data
☒	☐	Dual use research of concern
☒	☐	Plants

Methods

n/a	Involved in the study	
☒	☐	ChIP-seq
☒	☐	Flow cytometry
☒	☐	MRI-based neuroimaging

Plants

Seed stocks	No seed was used in manuscript.
Novel plant genotypes	No plant genotype was produced. No authentication procedures for each seed stock was generated
Authentication	Not relevant.
